# Effect of Levothyroxine on Older Patients With Subclinical Hypothyroidism: A Systematic Review and Meta-Analysis

**DOI:** 10.3389/fendo.2022.913749

**Published:** 2022-07-14

**Authors:** Chunyan Zhao, Yueqi Wang, Liu Xiao, Lin Li

**Affiliations:** Department of Nuclear Medicine, West China Hospital, Sichuan University, Chengdu, China

**Keywords:** older, levothyroxine, subclinical hypothyroidism, cholesterol, triglyceride, low-density lipoprotein cholesterol, apolipoprotein B

## Abstract

**Background:**

Subclinical hypothyroidism (SCH) is usually treated with levothyroxine, but there is controversy as to whether SCH should be treated, especially for older patients. The aim of the systematic review and meta-analysis was to evaluate whether levothyroxine has a beneficial or harmful effect on older patients with SCH.

**Methods:**

Databases including PubMed, Embase, Cochrane Library, Web of Science, Wanfang, Weipu and China National Knowledge Infrastructure were searched from inception until December 21, 2021. Subjects must be diagnosed with SCH, and older than or equal to 60 years of age. Interventions should be thyroid hormone therapy (e.g. levothyroxine). The literature was independently screened by 2 researchers. Statistical analysis was performed using RevMan5.3 software.

**Results:**

A total of 13 articles were included. Meta-analysis results showed that in older SCH patients, levothyroxine can significantly reduce cholesterol (TC) (*p* < 0.00001), triglyceride (TG) (*p* < 0.00001), low-density lipoprotein cholesterol (LDL-C) (*p* = 0.03) and apolipoprotein B (ApoB) (*p* < 0.00001). In addition, levothyroxine had no significant effect on bone mineral density, fatigue, hypothyroidism symptoms, quality of life, BMI, cognitive function, depression, blood pressure, etc. in older SCH patients, and also did not significantly increase the incidence of adverse events.

**Conclusions:**

Among older SCH patients, levothyroxine treatment may reduce TC, TG, LDL-C, and ApoB.

## Introduction

Subclinical hypothyroidism (SCH) is characterized by elevated thyroid stimulating hormone (TSH) and normal free thyroxin (FT4), which is a laboratory diagnosis (1). The symptoms of SCH are variable and may exhibit as fatigue, cold intolerance, lack of energy, etc., or may not exhibit any symptoms at all (2). The prevalence of SCH is about 4-20% and is higher in women and the older (2–4). SCH is usually treated with levothyroxine, but there is controversy as to whether and when SCH should be treated (2). American Thyroid Association (ATA) (5), American Association of Clinical Endocrinology (AACE) (6), and Brazilian Society of Endocrinology and Metabolism (BSEM) (7) all recommend to initiate levothyroxine treatment when TSH is greater than 10 mIU/L in patients with SCH. In addition, the guideline of European Thyroid Association (ETA) (8) indicates that levothyroxine treatment is recommended for younger severe SCH patients (<65 years, serum TSH >10 mU/l) with or without symptoms suggestive of hypothyroidism; while for younger mild SCH patients (<65 years old, TSH <10 mU/l) with symptoms suggestive of hypothyroidism and for older severe SCH patients (>70 years, serum TSH >10 mU/l) with symptoms of hypothyroidism or cardiovascular risk, levothyroxine therapy also should be considered. Follow up and observe were recommended in the rest of conditions. While neither British Thyroid Association (BTA) (9) nor Italian Association of Clinical Endocrinology (AME) (10) have specific criteria. Since older people are often accompanied by multiple organ dysfunction and underlying diseases, the treatment of older SCH patients should be individualized. Whether levothyroxine treatment will bring beneficial or harmful effect to older SCH patients, there is no definite conclusion.

Whether levothyroxine can provide benefit to older patients with SCH has been explored in several studies. Most studies did not find a significant effect of levothyroxine on older patients with SCH, such as bone density (11, 12), hypothyroidism symptoms and fatigue (13–15), depressive symptoms (16), cognitive function (17), cardiac function (18), ischemic heart disease (19), cardiovascular outcomes (20). However, a study (21) comparing old and young patients with SCH found that levothyroxine was effective in improving the quality of life of old patients. In addition, levothyroxine had also been found to improve lipid profile (22, 23) and preserve kidney function (24) in older patients with SCH. However, it was noteworthy that levothyroxine had also been found to increase mortality in older patients with SCH (25). This shows that it is controversial whether to initiate levothyroxine therapy in older patients with SCH.

The aim of this meta-analysis and systematic review was to analyze all published relevant studies and evaluate whether levothyroxine has a beneficial or harmful effect on older patients with SCH.

## Methods

**Figure 1:**
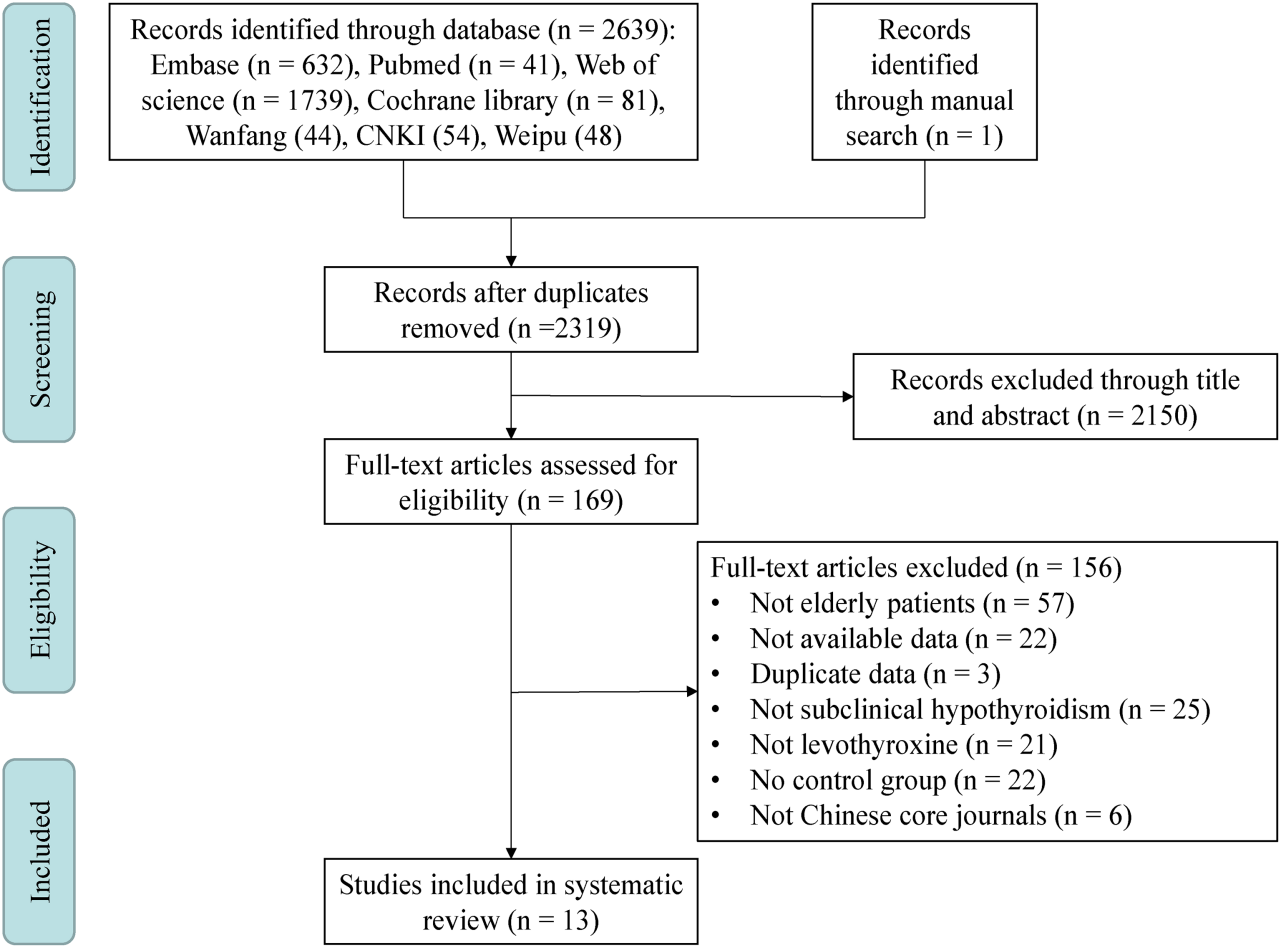
Meta-analysis flowchart. This meta-analysis included a total of four steps: Identification, Screening, Eligibility and Included. CNKI, China National Knowledge Infrastructure.

This study was consistent with the Preferred Reporting Items for a Systematic Review and Meta-analysis (PRISMA) guidelines (26), **Figure 1**.

### Study Selection

Studies included in the meta-analysis must meet the following criteria: 1) Subjects must be diagnosed with SCH that was defined as elevated TSH and normal FT4. 2) Subjects must be older than or equal to 60 years of age. 3) Interventions should be thyroid hormone therapy (e.g. levothyroxine).

Outcomes can be various aspects of older SCH patients, such as quality of life, cognitive function, depression, lipids profile, renal function, cardiovascular events, etc. We combined the same outcomes for meta-analysis.

### Data Sources and Search Strategy

We searched Embase, Web of Science, Cochrane Library, Pubmed and chinese databases including Wanfang, Weipu and China National Knowledge Infrastructure (CNKI) from inception until December 21, 2021. In order to ensure the quality of Chinese literature, we included articles from Chinese core journals and dissertation, which have undergone rigorous peer review and have a high degree of recognition. Subjects were human, and the published languages mainly included English and Chinese. To reduce selection bias, we also searched for articles published in German, Spanish, French, Korean and Japanese. The subject words included Hypothyroidism, and Thyroxine. The random words included Subclinical, Mild, Aged, Elder, Older, randomized controlled trial and so on (**Supplementary Text S1**). In order not to miss possible literature, we manually searched references of relevant papers obtained from systematic searches, as well as relevant conferences and registered clinical trials.

### Data Extraction and Quality Assessment

Two reviewers independently screened eligible articles and extracted relevant data from the included studies. If there was disagreement, a third reviewer would join to conclude the final results through a discussion. The first step was to screen the articles based on their titles and abstracts, and the second step was to read the full-text of selected articles for the final eligible literature.

The information extracted from each eligible literature included authors, year of publication, country, number and age of participants, follow-up time, outcomes and so on. For the ages of participants in some included literature, the mean and standard deviation were not used to record, we will use mathematical methods to convert. If a study measured outcomes at different follow-up time points, we would extract data from the time point most frequently used in the other studies with the same outcomes, or the data closest to that time point. For unavailable result data, we would get the correct data by sending an email to the author. Quality assessment was conducted according to the Cochrane Handbook for Systematic Reviews of Interventions Version 5.0.1. (Higgins JPT and Green S, n.d.).

### Statistical Analyses

The software RevMan5.3 was used to perform statistical analysis and generate forest plots in this meta-analysis study. The outcomes of some articles were continuous variables, such as quality of life, depression, etc., and standardized mean difference (SMD) was used as a summary analysis when measured with different tools, or using mean difference (MD) when the same tool was used. When the outcomes were dichotomous variables, such as cardiovascular events, odds ratio (OR) was used. The chi-square test and I^2^ test were used to assess the magnitude of the heterogeneity among the included studies. A fixed effects model was used if heterogeneity is not significant (P ≥ 0.1 or I^2^ ≤ 50%), otherwise, a random effects model was applied (P < 0.1 or I^2^ > 50%). We considered the effect to be statistically significant when the P value was less than 0.05.

A subgroup analysis was conducted. On the one hand, both prospective randomized controlled trials and retrospective case-control studies were included in the meta-analysis, and on the other hand, some included studies had significantly different follow-up times. In order to analyze the effects of the above factors, we excluded those retrospective studies and studies with large differences in follow-up time to analyze the outcomes again, and saw if there were differences.

## Results

### Selection and Characteristics of Studies

After systematic search of 7 databases and manual search, a total of 2640 papers were yielded. Three hundred and twenty-one duplicates were removed, 2150 papers were removed by title and abstract, 156 papers were removed after reading the full text, and finally 13 eligible papers were included (**Figure 1**). About 5000 participants were included in the meta-analysis and systematic review, all of whom were over 60 years of age. Individuals in the Stuber’s study (15) were all from the Stott’s study and had no new findings, so Stuber’s study were excluded. Individuals in the Gencer’s (18), Wildisen’s (16) and Gonzalez Rodriguez’s (12) studies, although also all from the Stott’s study, were continued to be included because these three articles analyzed different results. Individuals in the Mooijaart’s study (14) were only partially from the Stott’s study and another part from the Du Puy’s study (27), which was not an exact duplicate of the Stott’s study, so it was continued to be included in this mata-analysis. A total of 4 Chinese literatures (28–31) were included, of which 3 (28, 30, 31) were from Chinese core journals and the other (29) was a master’s dissertation, which was also included due to its low risk of bias. Except for English and Chinese literature, literature published in other languages (including German, Spanish, French, Korean and Japanese) in the above databases did not meet the inclusion criteria. In addition, no studies in the database of Cochrane library that met the inclusion criteria had unpublished data. The characteristics of the included studies were summarized in **Supplementary Table S1**.

### Qualitative Analysis

These included studies came from different countries, including the Switzerland, Ireland, Netherlands, United Kingdom, China, and Israel. Follow-up times ranged from 6 months to over 60 months, and most studies had a follow-up time of about one year. The main outcomes of eligible studies included bone density, tiredness, hypothyroid symptoms, quality of life, body mass index (BMI), cognitive function, depression, blood pressure, renal function, lipid profile and cardiovascular events. The criteria for SCH varied slightly from study to study, and some studies did not limit the upper limit of TSH. The risk of bias for all included studies were detailed in **Supplementary Table S2**. The overall risk of bias was “Low” for most studies (12–14, 16–18, 29). Two studies (19, 25) were retrospective case-control studies, and two other studies (24, 31) did not use randomization schemes, so their risk of bias was “High”. The remaining two articles (28, 30) did not clearly describe the experimental protocol, so the risk of bias was “Unclear”. Due to the small number of included studies for different outcomes, no funnel plot was drawn to describe publication bias.

### Meta-Analysis Results

**Figure 2:**
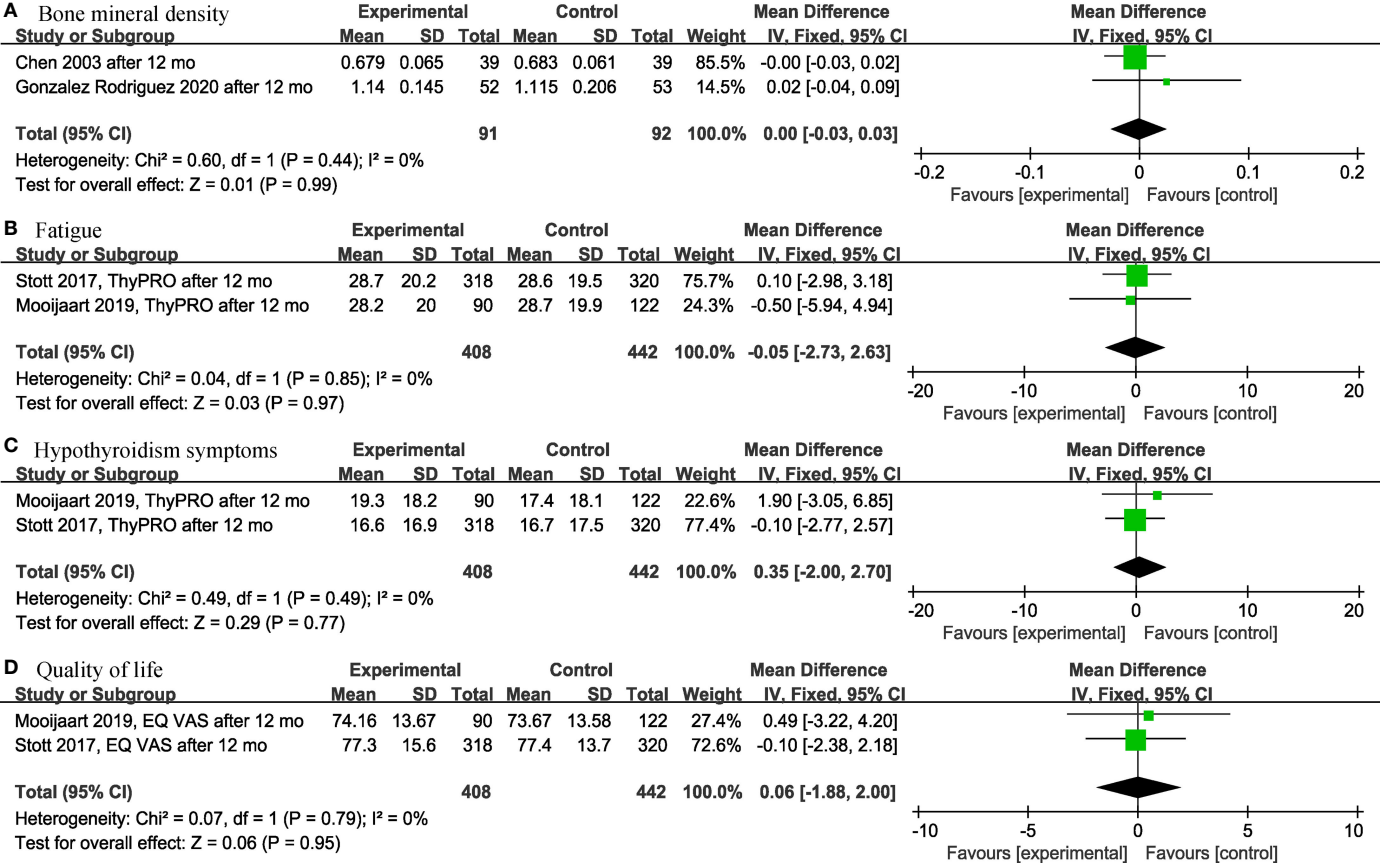
Forest plots depicting the effect of levothyroxine on the bone mineral density, fatigue, hypothyroidism symptoms and quality of life in older patients. **(A)** Association between levothyroxine and bone mineral density. **(B)** Association between levothyroxine and fatigue. **(C)** Association between levothyroxine and hypothyroidism symptoms. **(D)** Association between levothyroxine and quality of life.

**Figure 3:**
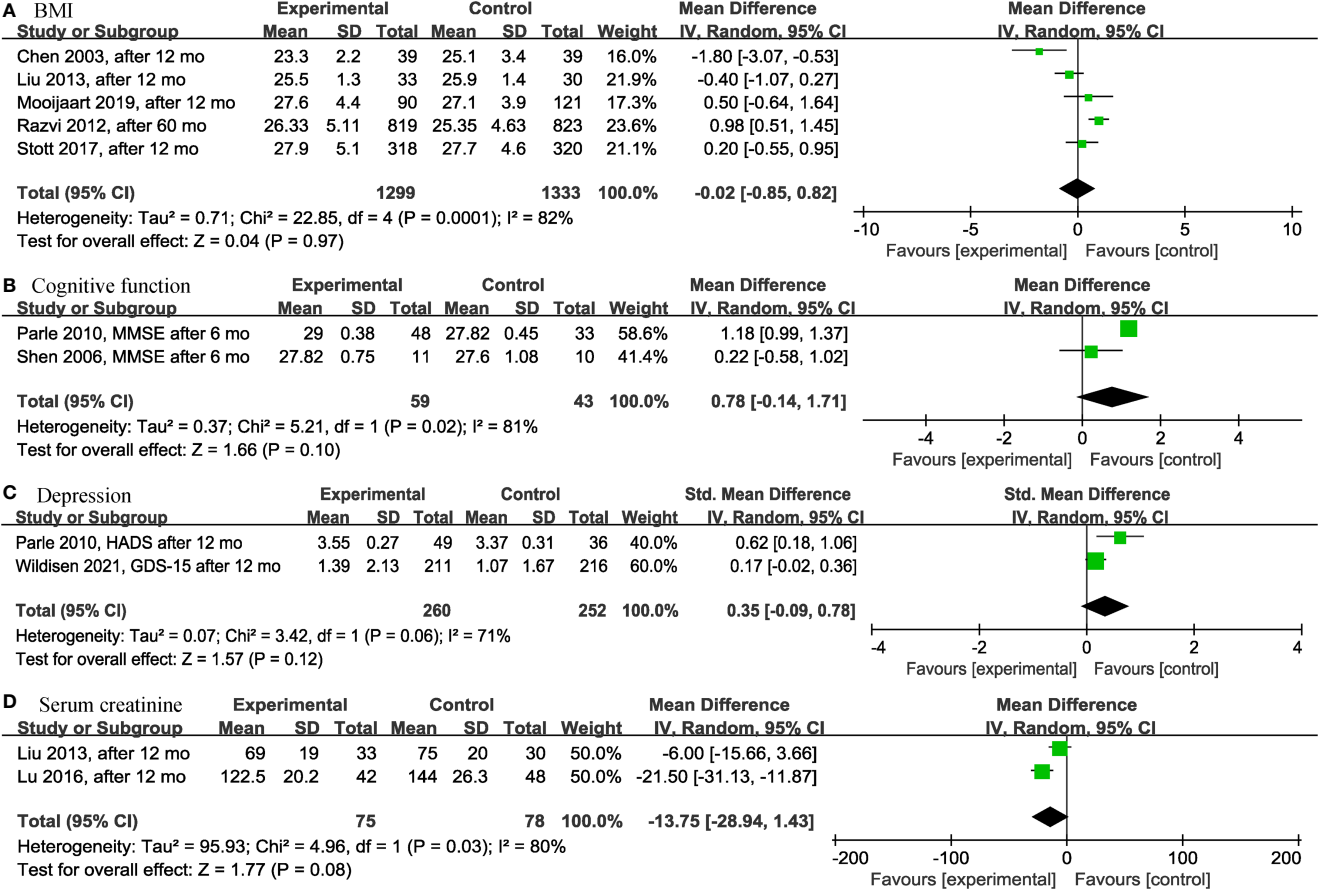
Forest plots depicting the effect of levothyroxine on the BMI, cognitive function, depression and serum creatinine in older patients. **(A)** Association between levothyroxine and BMI. **(B)** Association between levothyroxine and cognitive function. **(C)** Association between levothyroxine and depression. **(D)** Association between levothyroxine and serum creatinine. BMI, Body Mass Index.

**Figure 4:**
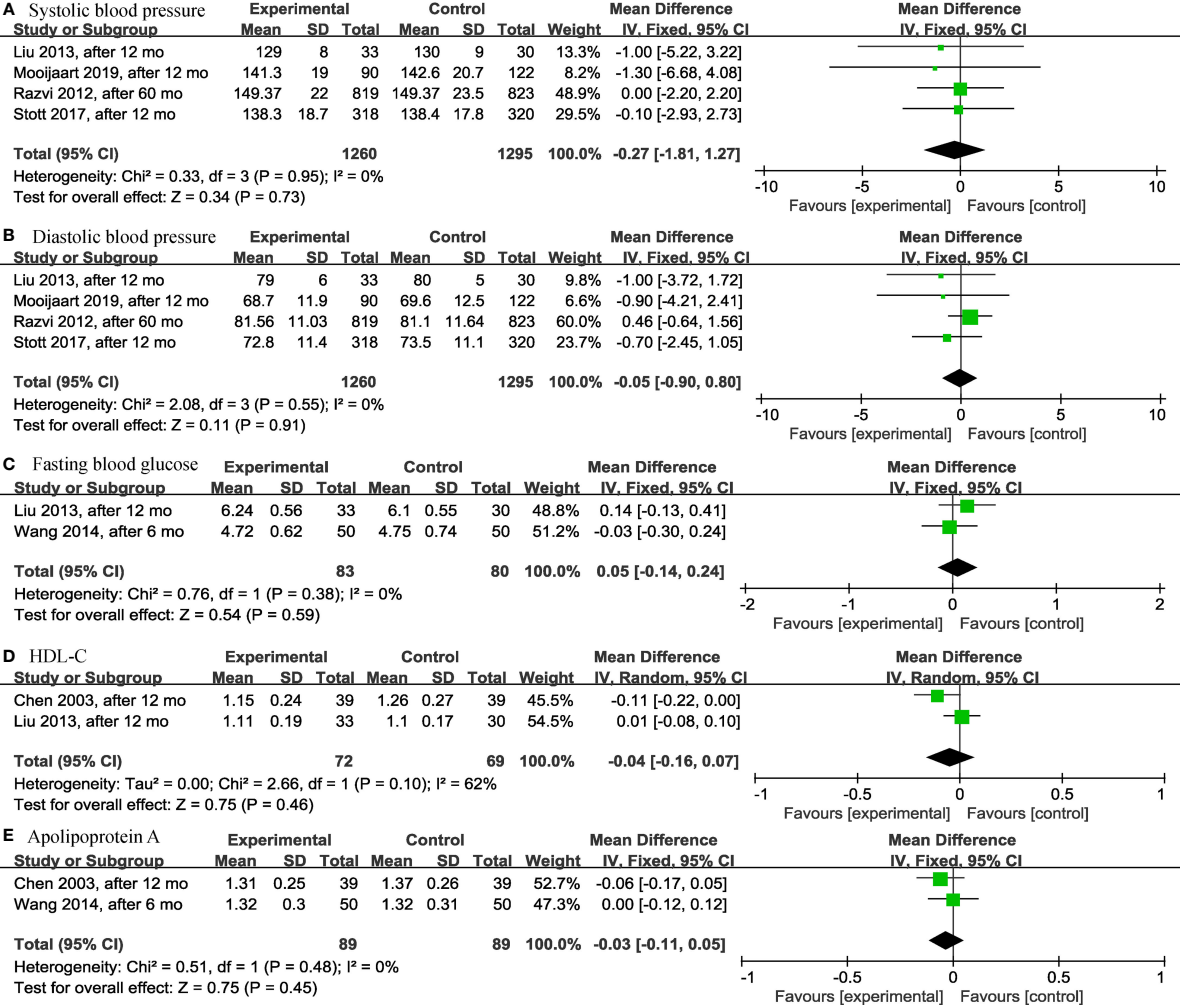
Forest plots depicting the effect of levothyroxine on the systolic blood pressure, diastolic blood pressure, fasting blood glucose, HDL-C and apolipoprotein A in older patients. **(A)** Association between levothyroxine and systolic blood pressure. **(B)** Association between levothyroxine and diastolic blood pressure. **(C)** Association between levothyroxine and fasting blood glucose. **(D)** Association between levothyroxine and HDL-C. **(E)** Association between levothyroxine and apolipoprotein A. HDL-C, High-Density Lipoprotein Cholesterol.

Among the continuous variable results, there were not significant differences regarding the effects of levothyroxine on bone mineral density (*p* = 0.99; MD = 0.00; 95% CI, -0.03 to 0.03; I^2^ = 0%) (12, 28) (**Figure 2A**), fatigue (*p* = 0.97; MD = -0.05; 95% CI, -2.73 to 2.63; I^2^ = 0%) (13, 14) (**Figure 2B**), hypothyroidism symptoms (*p* = 0.77; MD = 0.35; 95% CI, -2.00 to 2.70; I^2^ = 0%) (13, 14) (**Figure 2C**), quality of life (*p* = 0.95; MD = 0.06; 95% CI, -1.88 to 2.00; I^2^ = 0%) (13, 14) (**Figure 2D**), BMI (*p* = 0.97; MD = -0.02; 95% CI, -0.85 to 0.82; I^2^ = 82%) (13, 14, 19, 28, 30) (**Figure 3A**), cognitive function (*p* = 0.10; MD = 0.78; 95% CI, -0.14 to 1.71; I^2^ = 81%) (17, 29) (**Figure 3B**), depression (*p* = 0.12; SMD = 0.35; 95% CI, -0.09 to 0.78; I^2^ = 71%) (16, 17) (**Figure 3C**), serum creatinine (*p* = 0.08; MD = -13.75; 95% CI, -28.94 to 1.43; I^2^ = 80%) (24, 30) (**Figure 3D**), systolic blood pressure (*p* = 0.73; MD = -0.27; 95% CI, -1.81 to 1.27; I^2^ = 0%) (13, 14, 19, 30) (**Figure 4A**), diastolic blood pressure (*p* = 0.91; MD = -0.05; 95% CI, -0.90 to 0.80; I^2^ = 0%) (13, 14, 19, 30) (**Figure 4B**), fasting blood glucose (*p* = 0.59; MD = 0.05; 95% CI, -0.14 to 0.24; I^2^ = 0%) (30, 31) (**Figure 4C**), high-density lipoprotein cholesterol (HDL-C) (*p* = 0.46; MD = -0.04; 95% CI, -0.16 to 0.07; I^2^ = 62%) (28, 30) (**Figure 4D**) and apolipoprotein A (ApoA) (*p* = 0.45; MD = -0.03; 95% CI, -0.11 to 0.05; I^2^ = 0%) (28, 31) (**Figure 4E**). In the above analyses, both fatigue and hypothyroid symptoms were assessed using the Thyroid-Related Quality-of-Life Patient-Reported Outcome scale (ThyPRO), quality of life was assessed using the EuroQoL visual analogue scale (EQ VAS), and cognitive function was assessed using the Mini-Mental State Examination scale (MMSE). However, in 2 different studies, the Hospital Anxiety and Depression Scale (HADS) and 15-item Geriatric Depression Scale (GDS-15) were used to assess depression, respectively.

**Figure 5:**
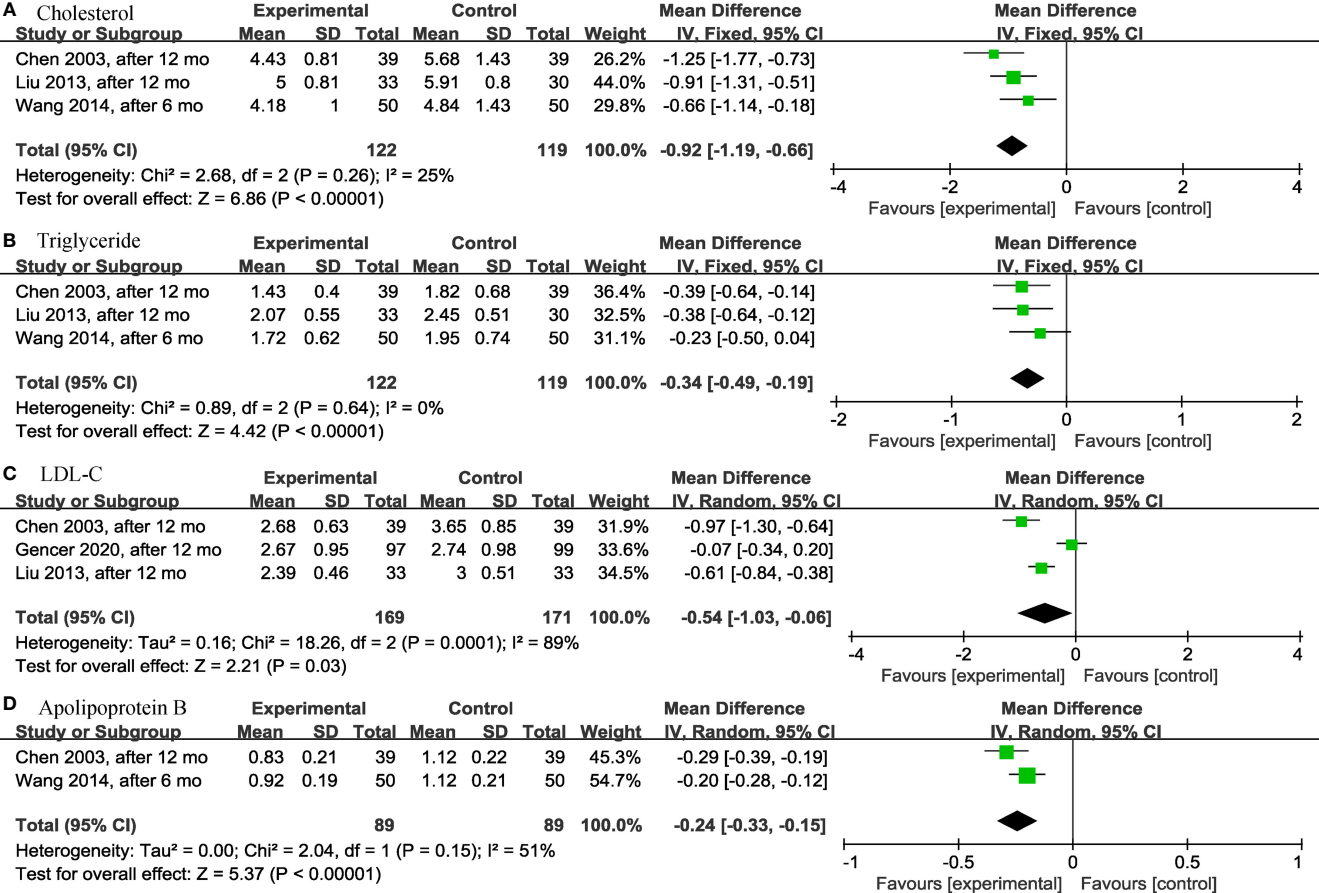
Forest plots depicting the effect of levothyroxine on the cholesterol, triglyceride, LDL-C and apolipoprotein B in older patients. **(A)** Association between levothyroxine and cholesterol. **(B)** Association between levothyroxine and triglyceride. **(C)** Association between levothyroxine and LDL-C. **(D)** Association between levothyroxine and apolipoprotein B. LDL-C, Low-Density Lipoprotein Cholesterol.

It was worth noting that, except for the above results, levothyroxine could have a significant effect on the lipid profile in older SCH patients, as shown below. Three articles (28, 30, 31) analyzed the effects of levothyroxine on cholesterol (TC) (*p* < 0.00001; MD = -0.92; 95% CI, -1.19 to -0.66; I^2^ = 25%) (**Figure 5A**) and triglyceride (TG) (*p* < 0.00001; MD = -0.34; 95% CI, -0.49 to -0.19; I^2^ = 0%) (**Figure 5B**) and showed significant differences in both. Levothyroxine also significantly reduced low-density lipoprotein cholesterol (LDL-C) (*p* = 0.03; MD = -0.54; 95% CI, -1.03 to -0.06; I^2^ = 89%) (**Figure 5C**) and apolipoprotein B (ApoB) (*p* < 0.00001; MD = -0.24; 95% CI, -0.33 to -0.15; I^2^ = 51%) (**Figure 5D**) in older SCH patients compared to controls, according to the analysis of 3 (18, 28, 30) and 2 (28, 31) articles, respectively.

**Figure 6:**
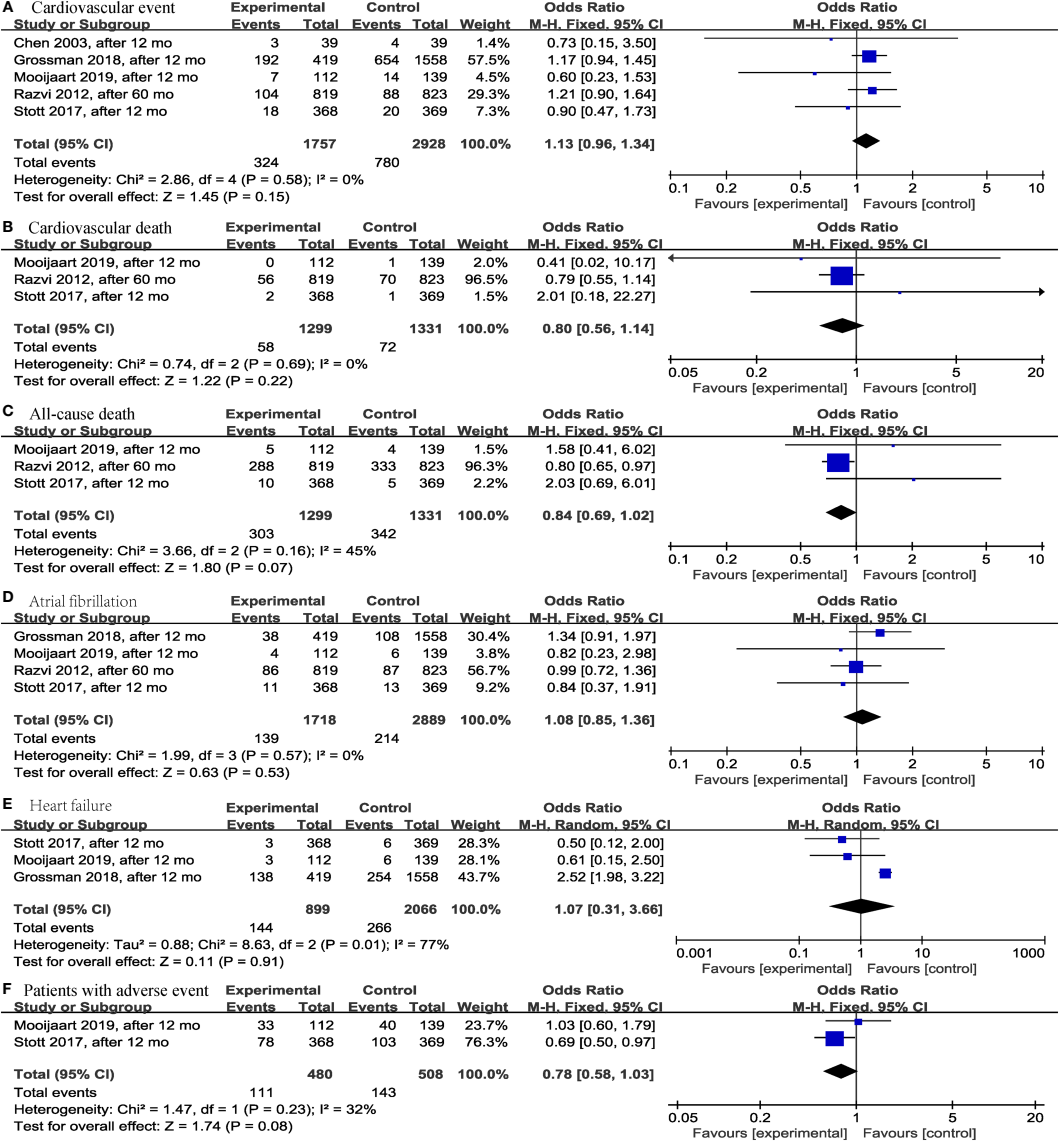
Forest plots depicting the effect of levothyroxine on the fatal or nonfatal cardiovascular event, cardiovascular death, all-cause death, atrial fibrillation, heart failure and number of patients with ≥1 serious adverse event in older patients. **(A)** Association between levothyroxine and fatal or nonfatal cardiovascular event. **(B)** Association between levothyroxine and cardiovascular death. **(C)** Association between levothyroxine and all-cause death. **(D)** Association between levothyroxine and atrial fibrillation. **(E)** Association between levothyroxine and heart failure. **(F)** Association between levothyroxine and number of patients with ≥1 serious adverse event.

Regarding adverse events that occurred during the study period, this meta-analysis did not find significant differences in the levothyroxine treatment group compared with the control group regarding fatal or nonfatal cardiovascular event (*p* = 0.15; OR = 1.13; 95% CI, 0.96 to 1.34; I^2^ = 0%) (13, 14, 19, 25, 28) (**Figure 6A**), cardiovascular death (*p* = 0.22; OR = 0.80; 95% CI, 0.56 to 1.14; I^2^ = 0%) (13, 14, 19) (**Figure 6B**), all-cause death (*p* = 0.07; OR = 0.84; 95% CI, 0.69 to 1.02; I^2^ = 45%) (13, 14, 19) (**Figure 6C**), atrial fibrillation (*p* = 0.53; OR = 1.08; 95% CI, 0.85 to 1.36; I^2^ = 0%) (13, 14, 19, 25) (**Figure 6D**), heart failure (*p* = 0.91; OR = 1.07; 95% CI, 0.31 to 3.66; I^2^ = 77%) (13, 14, 25) (**Figure 6E**), and number of patients with ≥1 serious adverse event (including new atrial fibrillation, heart failure, fracture, and new diagnosis of osteoporosis) (*p* = 0.08; OR = 0.78; 95% CI, 0.58 to 1.03; I^2^ = 32%) (13, 14) (**Figure 6F**) in older SCH patients.

### Subgroup and Sensitivity Analyses

To reduce the effect of different study protocols and different follow-up times on the results, we reanalyzed the results after removing retrospective case-control studies and studies with differences in follow-up times greater than six months. If these influences were not present, no re-analysis is required. Results of the reanalysis included BMI (*p* = 0.45; MD = -0.31; 95% CI, -1.10 to 0.48; I^2^ = 66%) (13, 14, 28, 30) (**Supplementary Figure S1A**), systolic blood pressure (*p* = 0.63; MD = -0.53; 95% CI, -2.68 to 1.63; I^2^ = 0%) (13, 14, 30) (**Supplementary Figure S1B**), diastolic blood pressure (*p* = 0.24; MD = -0.81; 95% CI, -2.15 to 0.54; I^2^ = 0%) (13, 14, 30) (**Supplementary Figure S1C**), fatal or nonfatal cardiovascular event (*p* = 0.33; OR = 0.78; 95% CI, 0.47 to 1.29; I^2^ = 0%) (13, 14, 28) (**Supplementary Figure S2A**), cardiovascular death (*p* = 0.92; OR = 1.09; 95% CI, 0.19 to 6.46; I^2^ = 0%) (13, 14) (**Supplementary Figure S2B**), all-cause death (*p* = 0.15; OR = 1.85; 95% CI, 0.80 to 4.27; I^2^ = 0%) (13, 14) (**Supplementary Figure S2C**), atrial fibrillation (*p* = 0.61; OR = 0.84; 95% CI, 0.42 to 1.67; I^2^ = 0%) (13, 14) (**Supplementary Figure S2D**), and heart failure (*p* = 0.24; OR = 0.55; 95% CI, 0.20 to 1.48; I^2^ = 0%) (13, 14) (**Supplementary Figure S2E**). These results did not change significantly from the original results.

## Discussion

This systematic review and meta-analysis found that levothyroxine treatment in older SCH patients was not associated with significant differences regarding bone mineral density, fatigue, hypothyroid symptoms, quality of life, BMI, cognitive function, depression, serum creatinine, blood pressure, fasting blood glucose, HDL-C, ApoA and adverse events (including fatal or nonfatal cardiovascular event, cardiovascular death, all-cause death, atrial fibrillation, heart failure, and number of patients with ≥1 serious adverse event). However, it is worth noting that levothyroxine significantly reduced TC, TG, LDL-C and ApoB in older SCH patients.

Compared with previous articles, some results were consistent with this meta-analysis. A meta-analysis (32) that included 21 RCTs found that nonpregnant adults with SCH did not benefit from levothyroxine treatment regarding the general quality of life, thyroid-related symptoms, fatigue, depressive symptoms, BMI, and blood pressure. Another meta-analysis (33) of 12315 individuals also found that SCH could increase the risk of depression, but levothyroxine treatment did not improve depression scores. Another two articles compared older SCH patients with normal (22) and hypothyroid patients (23) and found that levothyroxine significantly improved lipid profiles, such as lowering TC, TG, LDL, etc. These above results were consistent with the results of this article in older SCH patients. However, another article (34) including 30 SCH patients found that levothyroxine was able to significantly affect systolic blood pressure but not lipid profile, which is inconsistent with the results of this study. Therefore, more studies, especially for older SCH, are needed to obtain reliable conclusions.

Several studies (35, 36) had found that SCH significantly increased the incidence of adverse cardiovascular events, and it was controversial that levothyroxine treatment was effective in reducing the incidence of this adverse events. Some articles (19, 36) had found levothyroxine treatment to be effective in reducing adverse cardiovascular events in patients with SCH, but there were also studies (13, 37, 38) with opposite findings. In older SCH patients, most studies (14, 18) and this meta-analysis found that levothyroxine treatment did not significantly increase the incidence of negative events. Therefore, the safety of this treatment may need to be further evaluated by more high-quality studies before reliable conclusions can be drawn.

There were some limitations in this systematic review and meta-analysis. First, the number of included studies was too small, and fewer articles were available when analyzing a particular result. Second, some of the included articles were of low or unclear quality, expect more relevant studies with high quality to provide more reliable conclusions in the future. Third, the criteria for TSH were inconsistent in the included articles, and the range was too wide, new findings may be seen when dividing TSH into <10 mIU/L and ≥10 mIU/L in older SCH patients. Due to the small number of articles and limited available data, in this paper we did not perform further subgroup analysis on the TSH range. Fourth, the inclusion of Chinese literature may increase the difficulty of peer review, but it is necessary to include high-quality Chinese literature in this meta-analysis. Because levothyroxine treatment was found to improve lipid profiles after the inclusion of the Chinese literature, this meaningful conclusion may not be reached by relying only on the available English literature (a few English literature (22, 23) had similar conclusions but were not included in the analysis due to the different experimental protocols and control populations). Selection bias caused by language should be avoided. In addition, the only positive results of this meta-analysis were obtained from the analysis of Chinese journals. Although we have strictly controlled the quality of the included literature, the interpretation of the results should still be cautious, and more high-quality English literatures are still needed to verify the relevant conclusions in the future.

In summary, among older SCH patients, levothyroxine treatment may reduce TC, TG, LDL-C and ApoB. These results were important for reducing cardiovascular disease risk. Especially for older SCH patients with increased underlying diseases and reduced function of many body systems, levothyroxine therapy may be considered under the condition of ensuring safety. However, in clinical practice, the use of levothyroxine should still be cautious to avoid meaningless and excessive treatment.

## Data Availability Statement

The original contributions presented in the study are included in the article/**Supplementary Material**. Further inquiries can be directed to the corresponding author.

## Author Contributions

CZ contributed to the protocol development, literature research, data collection, data analysis, manuscript writing, and manuscript editing. The authors YW and LX performed literature research, data collection, and data analysis. The contributions of LL include protocol development, manuscript writing and manuscript editing. All authors contributed to the article and approved the submitted version.

## Funding

This study was supported by the “1.3.5 project for disciplines of excellence, West China Hospital, Sichuan University (ZYGD18016).”

## Conflict of Interest

The authors declare that the research was conducted in the absence of any commercial or financial relationships that could be construed as a potential conflict of interest.

## Publisher’s Note

All claims expressed in this article are solely those of the authors and do not necessarily represent those of their affiliated organizations, or those of the publisher, the editors and the reviewers. Any product that may be evaluated in this article, or claim that may be made by its manufacturer, is not guaranteed or endorsed by the publisher.
