# Supplementary material for: Effect of Levothyroxine on Older Patients With Subclinical Hypothyroidism: A Systematic Review and Meta-Analysis
Source: Front Endocrinol (Lausanne). 2022 Jul 14;13:913749. doi: 10.3389/fendo.2022.913749 (PMC9329610; doi:10.3389/fendo.2022.913749)
Supplement: Supplementary file 1 [file DataSheet_1.pdf]

Supplementary Table S1

Characteristics of studies included in the systematic review

Source	Country	Definition of Subclinical Hypothyroidism	No. of Participants	Age, Mean (SD)	Women, No. (%)	Intervention	Control	Follow-up Duration, mo	Outcomes
Chen, 2003	China	Elevated thyrotropin levels (>4.5 mU/L) and normal free thyroxine	78	65 (5.1)	62 (79.5)	Levothyroxine	Placebo	12	BMI, Bone mineral density, cardiovascular disease, Lipid profile
Shen, 2006	China	Elevated thyrotropin levels (>4.0 mIU/L) and normal free thyroxine	50	67.4 (4)	29 (58)	Levothyroxine	Placebo	6	HAMA, MMSE, CAMDEX
Parle, 2010	United Kingdom	Elevated thyrotropin levels (>5.5 mU/L) and free thyroxine from 9–20 pmol/liter	94	73.8 (5.8)	57 (60.6)	Levothyroxine	Placebo	6, 12	MMSE, MEAMS, SCOLP, Trail-Making Test, HADS
Razvi, 2012	United Kingdom	Elevated thyrotropin levels (5.01-10 mIU/L) and normal free thyroxine	1642	79.6 (6.3)	1315 (80.1)	Levothyroxine	No intervention	60+	Cardiovascular disease, mortality
Liu, 2013	China	Elevated thyrotropin levels (4.0-10.0 mIU/L) and normal free thyroxine	63	67 (4)	42 (66.7)	Levothyroxine + Conventional therapy	Conventional therapy	12	BMI, Blood pressure, Blood glucose, Lipid profile, Serum creatinine
Wang, 2014	China	Elevated thyrotropin levels and normal free thyroxine	100	69.2 (6.8)	76 (76)	Levothyroxine	Traditional Chinese medicine	6	Lipid profile, Blood glucose, Blood uric acid
Lu, 2016	China	Elevated thyrotropin levels (>4.5 mIU/L) and normal free thyroxine	90	78.2 (9.5)	29 (32.2)	Levothyroxine	No intervention	36	Serum creatinine, GFR
Stott, 2017	Netherlands, Switzerland,	Elevated thyrotropin levels (4.60–19.99 mIU/L) and normal free	737	74.4 (6.3)	396 (53.7)	Levothyroxine	Placebo	12	ThyPRO, EQ-5D, EQ VAS, Hand-grip strength, Blood

	Ireland, United Kingdom	thyroxine							pressure, BMI, Waist circumference, Adverse Events
Grossman, 2018	Israel	Elevated thyrotropin levels (4.2-10 mIU/L) and normal free thyroxine	1977	84 (11)	1516 (76.7)	Levothyroxine	No intervention	12+	Senile dementia, chronic renal failure, chronic obstructive lung disease, cardiovascular disease
Mooijaart, 2019	Netherlands, Switzerland, Ireland, United Kingdom	Elevated thyrotropin levels (4.6–19.9 mIU/L) and normal free thyroxine	251	84.6 (3.6)	118 (47.0)	Levothyroxine	Placebo	12	ThyPRO, EQ-5D, EQ VAS, Hand-grip strength, Blood pressure, BMI, Waist circumference, Adverse Events
Gonzalez Rodriguez, 2020	Switzerland	Elevated thyrotropin levels (≥4.6 and ≤19.9 mIU/L) and normal free thyroxine	196	74.3 (5.7)	89 (45.4)	Levothyroxine	Placebo	12	Bone mineral density, Trabecular bone score, bone turnover markers
Gencer, 2020	Switzerland	Elevated thyrotropin levels (4.60-19.99 mIU/L) and normal free thyroxine	185	74.1 (5.6)	87 (47.0)	Levothyroxine	Placebo	12	Cardiac function
Wildisen, 2021	Switzerland, Netherlands, Ireland	Elevated thyrotropin levels (4.6–19.9 mIU/L) and normal free thyroxine	427	74.5 (6.3)	239 (56.0)	Levothyroxine	Placebo	12	GDS-15, CESD-20

Abbreviations: Body Mass Index = BMI, Hamilton Anxiety Scale = HAMA, Mini-Mental State Examination = MMSE, Cambridge examination for mental disorders of the elderly = CAMDEX, Middlesex Elderly Assessment of Mental State = MEAMS, Speed and Capacity of Language Processing test = SCOLP, Hospital Anxiety and Depression Scale = HADS, Glomerular Filtration Rate = GFR, Thyroid-Related Quality-of-Life Patient-Reported Outcome = ThyPRO, EuroQoL Group 5-Dimension Self-Report Questionnaire = EQ-5D, EuroQoL visual analogue scale = EQ VAS, 15-item Geriatric Depression Scale = GDS-15, Center for Epidemiologic Studies Depression 20-item scale = CESD-20.
