# Supplementary material for: Effect of Levothyroxine on Older Patients With Subclinical Hypothyroidism: A Systematic Review and Meta-Analysis
Source: Front Endocrinol (Lausanne). 2022 Jul 14;13:913749. doi: 10.3389/fendo.2022.913749 (PMC9329610; doi:10.3389/fendo.2022.913749)
Supplement: Supplementary file 2 [file DataSheet_2.pdf]

Supplementary Table S2

Risk of bias assessment of the included randomized controlled trials

Author, year	Random sequence generation	Allocation concealment	Blinding of participants and personnel	Blinding of outcome assessment	Incomplete outcome data	Selective outcome reporting
Chen, 2003	Low	Unclear	Unclear	Unclear	Low	Low
Shen, 2006	Low	Low	Low	Low	Low	Low
Parle, 2010	Low	Low	Low	Low	Low	Low
Razvi, 2012	High	High	High	High	Low	Low
Liu, 2013	Low	Unclear	Unclear	Unclear	Low	Low
Wang, 2014	High	High	High	High	Low	Low
Lu, 2016	High	High	High	High	Low	Low
Stott, 2017	Low	Low	Low	Low	Low	Low
Grossman, 2018	High	High	High	High	Low	Low
Mooijaart, 2019	Low	Low	Low	Low	Low	Low
Gonzalez Rodriguez, 2020	Low	Low	Low	Low	Low	Low
Gencer, 2020	Low	Low	Low	Low	Low	Low
Wildisen, 2021	Low	Low	Low	Low	Low	Low
