# Supplementary material for: Effect of Levothyroxine on Older Patients With Subclinical Hypothyroidism: A Systematic Review and Meta-Analysis
Source: Front Endocrinol (Lausanne). 2022 Jul 14;13:913749. doi: 10.3389/fendo.2022.913749 (PMC9329610; doi:10.3389/fendo.2022.913749)
Supplement: Supplementary file 3 [file DataSheet_3.pdf]

Supplementary Text S1

We searched data bases including Embase, Web of Science, Cochrane Library, and Pubmed on December 21, 2021.

The search strategy was as follows:

#1 Hypothyroidism[MeSH Terms]

#2 (((((((((((((((Hypothyroidisms[Title/Abstract]) OR (Primary Hypothyroidism[Title/Abstract])) OR (Hypothyroidism, Primary[Title/Abstract])) OR (Primary Hypothyroidisms[Title/Abstract])) OR (Thyroid-Stimulating Hormone Deficiency[Title/Abstract])) OR (Deficiency, Thyroid-Stimulating Hormone[Title/Abstract])) OR (Hormone Deficiency, Thyroid-Stimulating[Title/Abstract])) OR (Thyroid Stimulating Hormone Deficiency[Title/Abstract])) OR (Thyroid-Stimulating Hormone Deficiencies[Title/Abstract])) OR (TSH Deficiency[Title/Abstract])) OR (Deficiency, TSH[Title/Abstract])) OR (TSH Deficiencies[Title/Abstract])) OR (Secondary Hypothyroidism[Title/Abstract])) OR (Hypothyroidism, Secondary[Title/Abstract])) OR (Secondary Hypothyroidisms[Title/Abstract])) OR (Central Hypothyroidism[Title/Abstract])) OR (Central Hypothyroidisms[Title/Abstract])) OR (Hypothyroidism, Central[Title/Abstract])

#3 #1 OR #2

#4 (((((Asymptomatic[Title/Abstract]) OR (Mild[Title/Abstract])) OR (Subclinical[Title/Abstract])) OR (Sub-clinical[Title/Abstract])) OR (Subclin*[Title/Abstract])) OR (Sub-clin*[Title/Abstract])

#5 #3 AND #4

#6 ((Thyroxine[MeSH Terms]) OR (O-(4-Hydroxy-3,5-diiodophenyl)-3,5-diiodotyrosine[Title/Abstract])) OR (Thyroxin[Title/Abstract])) OR (3,5,3',5'-Tetraiodothyronine[Title/Abstract])) OR (T4 Thyroid Hormone[Title/Abstract])) OR (Thyroid Hormone, T4[Title/Abstract])) OR (Synthrox[Title/Abstract])) OR (Levothyroxine Sodium[Title/Abstract])) OR (Sodium Levothyroxine[Title/Abstract])) OR (Thyrax[Title/Abstract])) OR (Tiroidine[Title/Abstract])) OR (Tiroxina Leo[Title/Abstract])) OR (Unithroid[Title/Abstract])) OR (Eferox[Title/Abstract])) OR (Eltroxin[Title/Abstract])) OR (Thevier[Title/Abstract])) OR (Eltroxine[Title/Abstract])) OR (Euthyrox[Title/Abstract])) OR (Eutirox[Title/Abstract])) OR (L-Thyrox[Title/Abstract])) OR (L Thyrox[Title/Abstract])) OR (L-Thyroxin beta[Title/Abstract])) OR (L Thyroxin beta[Title/Abstract])) OR (L-Thyroxin Henning[Title/Abstract])) OR (L Thyroxin Henning[Title/Abstract])) OR (Levothyroxine[Title/Abstract])) OR (O-(4-Hydroxy-3,5-diiodophenyl) 3,5-diiodo-L-tyrosine[Title/Abstract])) OR (L-Thyroxine[Title/Abstract])) OR (L Thyroxine[Title/Abstract])) OR (L-3,5,3',5'-Tetraiodothyronine[Title/Abstract])) OR (Levoxine[Title/Abstract])) OR (Levoxyl[Title/Abstract])) OR (Lévothyrox[Title/Abstract])) OR (L-Thyroxine Roche[Title/Abstract])) OR (L Thyroxine Roche[Title/Abstract])) OR

(Levo-T[Title/Abstract])) OR (Levo T[Title/Abstract])) OR (Levothroid[Title/Abstract])) OR (Novothyral[Title/Abstract])) OR (Berlthyrox[Title/Abstract])) OR (Dexnon[Title/Abstract])) OR (Novothyrox[Title/Abstract])) OR (Oroxine[Title/Abstract])) OR (Synthroid[Title/Abstract])) OR (Levothyroxin Deladande[Title/Abstract])) OR (Levothyroxin Delalande[Title/Abstract])) OR (Levothyroid[Title/Abstract])

#7 #5 AND #6

#8 (((Aged[Title/Abstract]) OR (Elder[Title/Abstract])) OR (Older[Title/Abstract])) OR (Elderly[Title/Abstract])

#9 #7 AND #8

#10 (((((randomized controlled trial[Publication Type]) OR (randomized[Title/Abstract])) OR (placebo[Title/Abstract])) OR (meta-analysis[Publication Type])) OR (meta analysis[Title/Abstract])) OR (meta analysis[MeSH Terms])

#11 #9 AND #10
