# Supplementary material for: Effect of Levothyroxine on Older Patients With Subclinical Hypothyroidism: A Systematic Review and Meta-Analysis
Source: Front Endocrinol (Lausanne). 2022 Jul 14;13:913749. doi: 10.3389/fendo.2022.913749 (PMC9329610; doi:10.3389/fendo.2022.913749)

A BMI

Study or Subgroup	Experimental Mean	Experimental SD	Experimental Total	Control Mean	Control SD	Control Total	Weight	Mean Difference IV, Random, 95% CI
Chen 2003, after 12 mo	23.3	2.2	39	25.1	3.4	39	19.4%	-1.80 [-3.07, -0.53]
Liu 2013, after 12 mo	25.5	1.3	33	25.9	1.4	30	30.4%	-0.40 [-1.07, 0.27]
Mooijaart 2019, after 12 mo	27.6	4.4	90	27.1	3.9	121	21.4%	0.50 [-0.64, 1.64]
Stott 2017, after 12 mo	27.9	5.1	318	27.7	4.6	320	28.7%	0.20 [-0.55, 0.95]
**Total (95% CI)**			**480**			**510**	**100.0%**	**-0.31 [-1.10, 0.48]**

Heterogeneity: Tau² = 0.42; Chi² = 8.92, df = 3 (P = 0.03); I² = 66%
Test for overall effect: Z = 0.76 (P = 0.45)

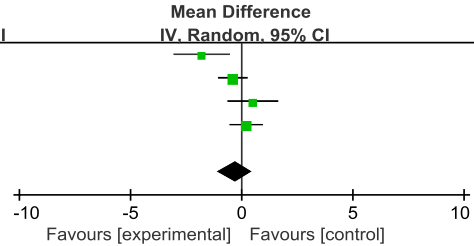

B Systolic blood pressure

Study or Subgroup	Experimental Mean	Experimental SD	Experimental Total	Control Mean	Control SD	Control Total	Weight	Mean Difference IV, Fixed, 95% CI
Liu 2013, after 12 mo	129	8	33	130	9	30	26.1%	-1.00 [-5.22, 3.22]
Mooijaart 2019, after 12 mo	141.3	19	90	142.6	20.7	122	16.1%	-1.30 [-6.68, 4.08]
Stott 2017, after 12 mo	138.3	18.7	318	138.4	17.8	320	57.9%	-0.10 [-2.93, 2.73]
**Total (95% CI)**			**441**			**472**	**100.0%**	**-0.53 [-2.68, 1.63]**

Heterogeneity: Chi² = 0.21, df = 2 (P = 0.90); I² = 0%
Test for overall effect: Z = 0.48 (P = 0.63)

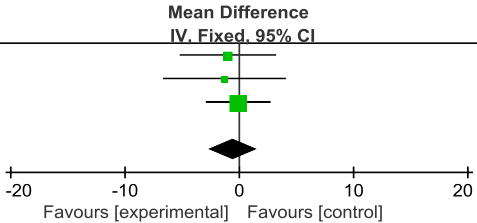

C Diastolic blood pressure

Study or Subgroup	Experimental Mean	Experimental SD	Experimental Total	Control Mean	Control SD	Control Total	Weight	Mean Difference IV, Fixed, 95% CI
Liu 2013, after 12 mo	79	6	33	80	5	30	24.4%	-1.00 [-3.72, 1.72]
Mooijaart 2019, after 12 mo	68.7	11.9	90	69.6	12.5	122	16.4%	-0.90 [-4.21, 2.41]
Stott 2017, after 12 mo	72.8	11.4	318	73.5	11.1	320	59.2%	-0.70 [-2.45, 1.05]
**Total (95% CI)**			**441**			**472**	**100.0%**	**-0.81 [-2.15, 0.54]**

Heterogeneity: Chi² = 0.04, df = 2 (P = 0.98); I² = 0%
Test for overall effect: Z = 1.18 (P = 0.24)

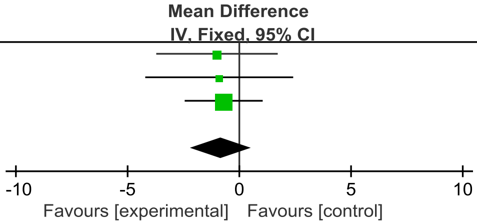

Supplement: Supplementary file 4 [file DataSheet_4.pdf]
