# Supplementary figures and images for: Effect of Levothyroxine on Older Patients With Subclinical Hypothyroidism: A Systematic Review and Meta-Analysis
Source: Front Endocrinol (Lausanne). 2022 Jul 14;13:913749. doi: 10.3389/fendo.2022.913749 (PMC9329610; doi:10.3389/fendo.2022.913749)

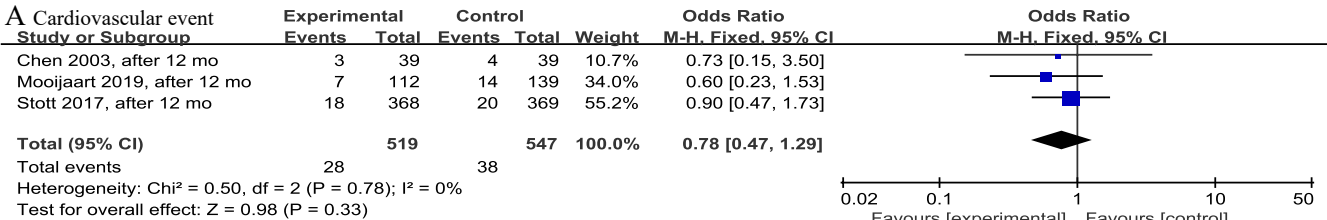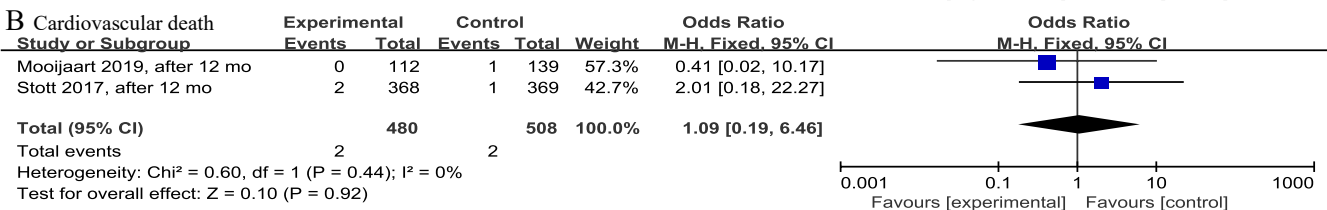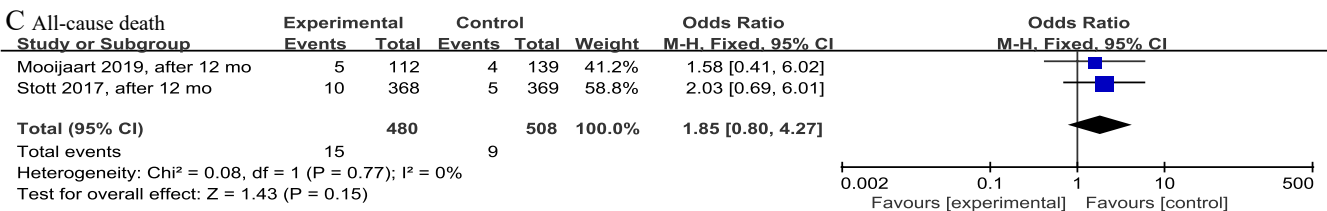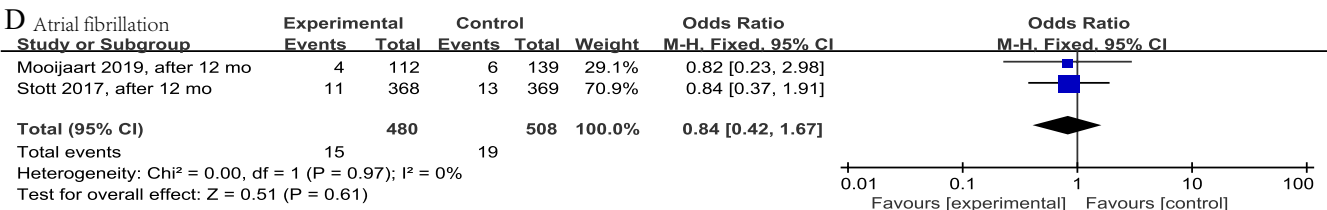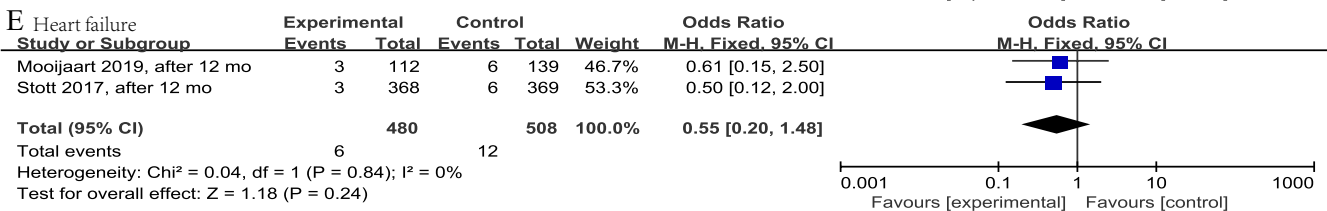

Supplement: Supplementary file 5 [file DataSheet_5.pdf]
